# Biomarkers in Exhaled Breath Condensate and Serum of Chronic Obstructive Pulmonary Disease and Non-Small-Cell Lung Cancer

**DOI:** 10.1155/2013/578613

**Published:** 2013-08-01

**Authors:** Mann Ying Lim, Paul S. Thomas

**Affiliations:** ^1^Inflammation and Infection Research Centre, Faculty of Medicine, University of New South Wales, Sydney, NSW 2031, Australia; ^2^Department of Respiratory Medicine, Prince of Wales Hospital, Randwick, Sydney, NSW 2031, Australia

## Abstract

Chronic obstructive pulmonary disease (COPD) and lung cancer are leading causes of deaths worldwide which are associated with chronic inflammation and oxidative stress. Lung cancer, in particular, has a very high mortality rate due to the characteristically late diagnosis. As such, identification of novel biomarkers which allow for early diagnosis of these diseases could improve outcome and survival rate. Markers of oxidative stress in exhaled breath condensate (EBC) are examples of potential diagnostic markers for both COPD and non-small-cell lung cancer (NSCLC). They may even be useful in monitoring treatment response. In the serum, S100A8, S100A9, and S100A12 of the S100 proteins are proinflammatory markers. They have been indicated in several inflammatory diseases and cancers including secondary metastasis into the lung. It is highly likely that they not only have the potential to be diagnostic biomarkers for NSCLC but also prognostic indicators and therapeutic targets.

## 1. Introduction

Chronic obstructive pulmonary disease (COPD) and lung cancer are the leading causes of deaths worldwide which are associated with cigarette smoking. COPD is a preventable and treatable disease characterised by progressive, irreversible airflow obstruction resulting from chronic airway inflammation [1–3]. It is responsible for 5.8% of all deaths (3.28 million deaths in 2008) and expected to become the third leading cause of death by 2030 [4]. Lung cancer, on the other hand, is defined as cancer which arises from cells of respiratory epithelium [5]. It has been the global leading cause of cancer death (approximately 1.8 million deaths per year) since 1985 [5], accounting for 12.4% of total new cancer cases diagnosed [5] and almost as many deaths as those from prostate, breast, and colon cancer combined [6]. The majority (85%) of lung cancer is non-small-cell lung cancer (NSCLC), and it can be further divided into adenocarcinoma, squamous cell carcinoma, and large cell carcinoma comprising 38.5%, 20%, and 2.9% of all lung cancer cases, respectively [5]. 

**Figure 1:**
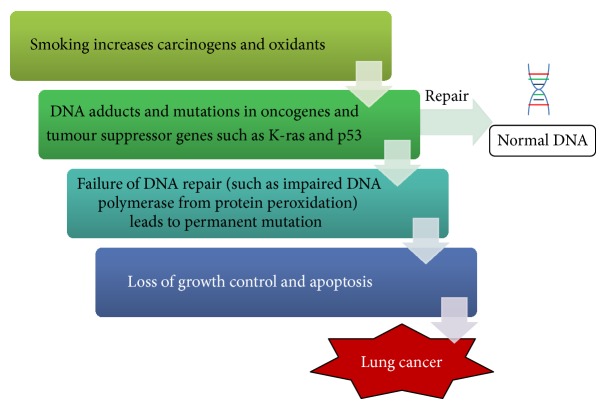
Stepwise progression towards lung cancer. Oxidants in cigarette smoking induce inflammation which subjects DNA to mutations. The failure to repair damaged DNA in critical coding regions causes cell proliferation and lung cancer.

Despite significant advances in 5-year survival rates of other cancers, that of lung cancer remains low at 15.6% (compared to 66% for colon cancer, 94% for melanoma, 90% for breast cancer, and 100% for prostate cancer) [6, 7]. Even more disappointingly, >52% of the patients have distant metastases (stage IV) at the time of diagnosis with a resultant 5-year survival of <3.6% (Figure 1) [5]. This is in stark contrast to the 60%–80% 5-year survival rate for patients with stage I lung cancer [8]. Patients usually present late as lung cancer is silent early in its course of disease and the symptoms are often nonspecific, thereby mistakenly attributed to ageing or smoking [9]. Furthermore, screening procedures such as sputum cytology and chest X-rays have failed to decrease mortality [10, 11]. Although screening CT scans increase the detection rate of early-stage lung cancer or small noncalcified nodules, the effect on mortality rate is still being evaluated, and the benefits need to be weighed against risks including radiation exposure, false positives, and overdiagnosis [12–16]. 

Much research has thus been directed towards the hope of finding new, simple, and minimally invasive biomarkers of early diagnosis or screening for COPD and lung cancer. Exhaled breath condensate and serum samples are two such examples.

## 2. Linking COPD and Lung Cancer

It is well established that both COPD and lung cancer are usually due to tobacco smoking [17–22]. The majority (90%) of lung cancers are associated with tobacco smoking [1], and smokers have a 2–30-fold increase in the risk of developing lung cancer [21, 23]. 

Apart from smoking, COPD is itself an independent risk factor [5, 7, 18, 24] which elevates the risk of lung cancer by 4.5 times [1, 7], and 1% of COPD patients develop lung cancer each year [18] while 40%–70% of lung cancer patients also have COPD [19, 22, 25, 26]. Furthermore, a positive correlation exists between the extent of airflow limitation and incidence of lung cancer [3, 18]. Even emphysema in never smokers (such as that of *α* 1-antitrypsin deficient carriers) also carries an elevated risk of lung cancer by 2.4-fold [22]. 

It is also known that COPD patients are at increased risk of developing squamous cell carcinoma with a worse prognosis as they not only develop higher grade tumours but also suffer from a higher rate of recurrence [1, 18, 27, 28].

## 3. Chronic Inflammation and Oxidative Stress

COPD and lung cancer are both associated with chronic inflammation and oxidative stress, [3, 19, 29, 30] in which oxidants, inflammatory mediators, and antioxidants are key players.

### 3.1. Oxidants

Oxidants can be generated exogenously or endogenously. Exogenous sources of oxidants include tobacco smoke, infections, and pollutants (such as ozone and nitrogen dioxide) [31, 32]. Of these sources, cigarette smoking is a major contributor as one puff contains up to 10^15^ oxidants particles and approximately 4700 different compounds [19, 31, 33]. Endogenously, oxidants are not only produced from the lung epithelial cells during respiration but also inflammatory mediators are released from cells such as neutrophils, eosinophils, and activated macrophages during inflammation [34–37]. They are generated through the mitochondrial electron transport chain during respiration and peroxidase enzymes such as myeloperoxidase (MPO), eosinophil peroxidase (EPO), and heme peroxidase during inflammation. 

Under normal physiological conditions, oxidants have a role in growth regulation, intracellular signaling, and host defence (inflammation) against infection [38]. They comprise reactive oxygen species (ROS) or reactive nitrogen species (RNS). Examples of ROS include superoxide (^•^O_2_^−^), hydroxyl radicals (^•^OH), and hydrogen peroxide (H_2_O_2_) while RNS includes nitric oxide (^•^NO), nitrogen dioxide, and peroxynitrite (ONOO^−^) [32]. Superoxide can be dismutated to hydrogen peroxide. In the presence of redox-active transition metals such as iron or copper, highly unstable hydroxyl radical can be generated from hydrogen peroxide in a reaction known as the Fenton reaction. Meanwhile, nitric oxide readily reacts with ROS to form peroxynitrite which breaks down into nitrite (NO_2_^−^) and nitrate (NO_3_^−^).

Reactive species are very unstable and potentially damaging as their unpaired electrons can exert injurious effects by oxidising DNA, proteins, and lipids [37, 39].

### 3.2. Inflammation and Oxidative Stress

The introduction of oxidants into the lung from tobacco smoking activates the innate immune cells such as lung epithelial cells whereby damage-associated molecular patterns (DAMPs) are released from injured cells [40]. Following this event, inflammation, which is the body's normal response to combat toxicants, is triggered [41–44] by the activation of transcription factor nuclear factor-*κ*B (NF-*κ*B) and activator protein 1 (AP-1) in airway epithelial cells and macrophages [29, 45]. The activated transcription factors are then responsible for the transcription of downstream inflammatory cytokines such as interleukin-6 (IL-6), interleukin-8 (IL-8), and tumour necrosis factor *α* (TNF-*α*) [34, 45–47]. The resultant elevated cytokine levels then attract more neutrophils and macrophages to augment inflammation (Figure 3) [29, 32, 45]. The degree of inflammation as evident by the infiltration of inflammatory cells correlates with disease severity [1, 19, 29].

**Figure 2:**
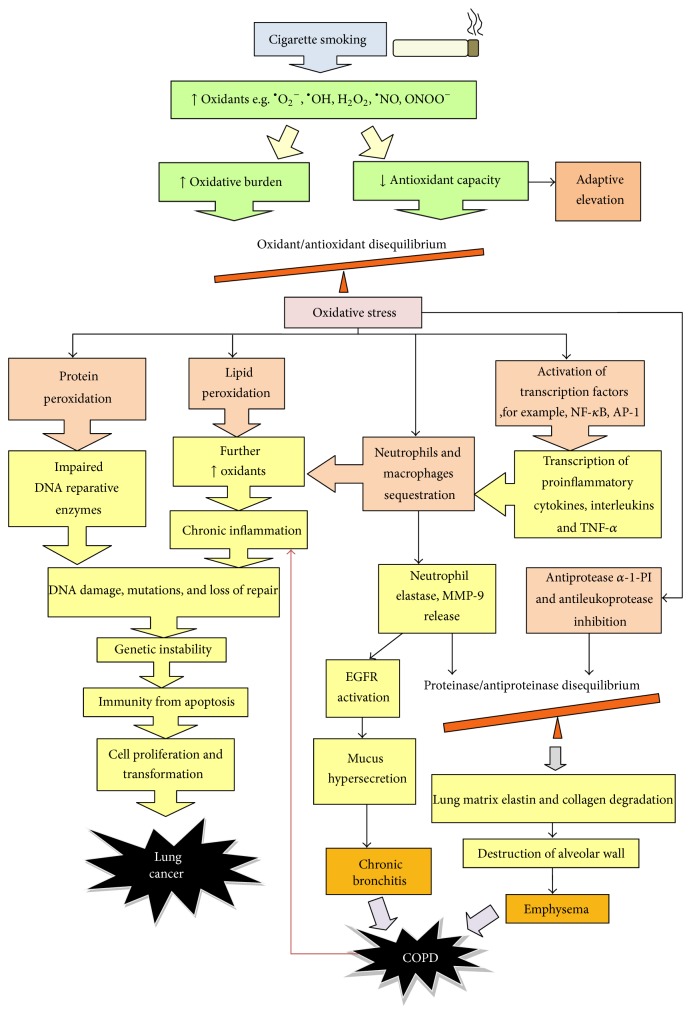
Smoking is the major cause of COPD and lung cancer. Oxidants in cigarette smoking are not only a direct cause of lung cancer by DNA damage through protein and lipid peroxidation but also an indirect cause by triggering inflammation. While products of recruited inflammatory mediators cause COPD by degrading lung matrix and promoting mucus hypersecretion, COPD is itself a disease of chronic inflammation which promotes tumorigenesis.

Following recruitment, neutrophils and macrophages release neutrophil elastase and matrix metalloproteinases-9 which are proteases that degrade lung matrix elastin and collagen [29, 32, 36, 44]. In addition, antiproteinases such as *α*-1-protease-inhibitor (*α*-1-PI) and antileukoprotease [32] are inactivated by oxidants [34], leading to a proteinase/antiproteinase imbalance which destroys the alveolar wall, causing airspace enlargement (emphysema) in COPD (Figure 2) [19, 29]. 

In addition, injuries during inflammation also lead to goblet cell hyperplasia and squamous metaplasia. This impairs mucociliary clearance, and inflammatory mediators accumulate in the airways as a result, which again amplifies inflammation [1]. The activation of epithelial growth factor receptor (EGFR) in response to neutrophil elastase and oxidative stress is another reason for mucus hypersecretion [1]. 

Apart from initiating inflammation, oxidants also readily attack polyunsaturated fatty acids of cell membranes to form lipid peroxidation products (LPPs) such as hydroperoxides, endoperoxides, and aldehydes including ethane, pentane, isoprostanoids, malondialdehyde, and 4-hydroxy-2nonenal which are even more reactive [31, 32, 46, 48]. Lipid peroxidation destroys cells by damaging cell membrane [31], and LPPs also react with DNA to cause genomic instability [48].

### 3.3. Oxidant/Antioxidant Disequilibrium

Under normal conditions, oxidants are counterbalanced by antioxidants which consist of enzymes (superoxide dismutase, catalase, glutathione peroxidase, and glutathione-S-transferase) and nonenzymatic free radical scavengers (glutathione, cysteine, thioredoxin, vitamins C and E, beta-carotene, and uric acid) [46]. 

In response to elevated levels of oxidants, local antioxidants such as superoxide dismutase, catalase, glutathione associated enzymes, and manganese superoxide dismutase may increase in an attempt to counter the insult [49–51]. The continuous introduction of oxidants from smoking, however, persistently exposes the lung parenchyma to raised oxidant levels, causing chronic inflammation. This exhausts the buffering capacity of antioxidants, giving rise to an oxidant/anti-oxidant disequilibrium which leads to oxidative stress and cellular damage [32, 42, 45, 52]. 

### 3.4. Chronic Inflammation and DNA Damage

Chronic inflammation increases cell turnover and replication errors [19, 42, 44, 53–55]. Replication errors which can occur include adduct formation, single or double stranded DNA breaks, promoter hypermethylation, sequence mutations, base insertions and deletions, translocations, microsatellite alterations, oncogene activation, and tumour suppressor gene inactivation [1, 46, 48, 56–59]. For smokers with lung cancer, mutations commonly occur in the K-ras oncogene and p53 tumour suppressor genes as well as there being p16 promoter hypermethylation [60–65]. The DNA mutations may confer on the cells a survival advantage by allowing cells to escape from apoptosis thereby proliferating uncontrollably [5, 62, 64]. 

Proofing mechanisms of DNA may attempt to repair or remove the damaged DNA via direct repair, double-strand break repair, cross-link repair, nucleotide excision, and base excision [1]. When damaged beyond repair, the cell usually undergoes apoptosis [5]. However, if any of the steps of reparation fail, or that damage to DNA is too extensive, permanent mutations may occur in the DNA, resulting in oncogenesis.

Apart from direct DNA damage, oxidants also promote tumorigenesis by direct reaction with proteins (protein peroxidation) to impair DNA reparative enzymes such as DNA polymerase [58]. 

## 4. Exhaled Breath Condensate

Exhaled breath condensate (EBC) is the cooling of exhaled gas to gain insight into the composition of extracellular lining fluid (ELF) and soluble exhaled gases [35, 66–68]. Compounds which have been measured include lipid peroxidation products, products of nitrogen oxide metabolism, hydrogen ions, hydrogen peroxide, cytokines, proteins, and DNA [69–71]. 

EBC has several advantages as an investigational technique. It is noninvasive (unlike bronchoalveolar lavage), inexpensive, easy to collect, and also easily repeatable without causing airway inflammation or dysfunction (unlike bronchoalveolar lavage, transbronchial biopsy or induced sputum analysis) [66, 67, 72, 73]. Furthermore, EBC collection devices are portable, do not induce any patient discomfort, and can thus be used in children and mechanically ventilated patients [67, 71, 74–76]. 

EBC has the potential to be employed in the screening and diagnosis of COPD and lung cancer, disease phenotyping, exacerbations, and treatment response monitoring as well as disease severity measuring and prognosis indicating [66, 68, 72, 77]. For instance, the use of EBC to measure lung antioxidant capacity could enable the monitoring of a response to antioxidant or anti-inflammatory treatment [78, 79]. It may also allow early anti-inflammatory treatment before the development of symptoms and lung function decline in COPD [78, 79]. 

EBC, however, has a number of limitations which include dilution by water vapour, nonsite specificity, saliva contamination and variable reproducibility. With >99.9% of EBC comprising water vapour [67], concentrations of the mediators of interest can sometimes be close to or below the detection limit of the appropriate assays; thus, assays of sufficient sensitivity are needed to effectively measure biomarkers in EBC [71, 77]. There is currently no standardised assessment of EBC dilution, but such issues can in part be overcome by correcting the dilution with urea, total protein, or cation concentration and conductivity of lyophilized EBC [71, 80, 81]. EBC dilution may also influence the pH. It is thus important to deaerate the sample and monitor the dilution and buffering capacity of EBC when measuring pH [82]. 

**Figure 3:**
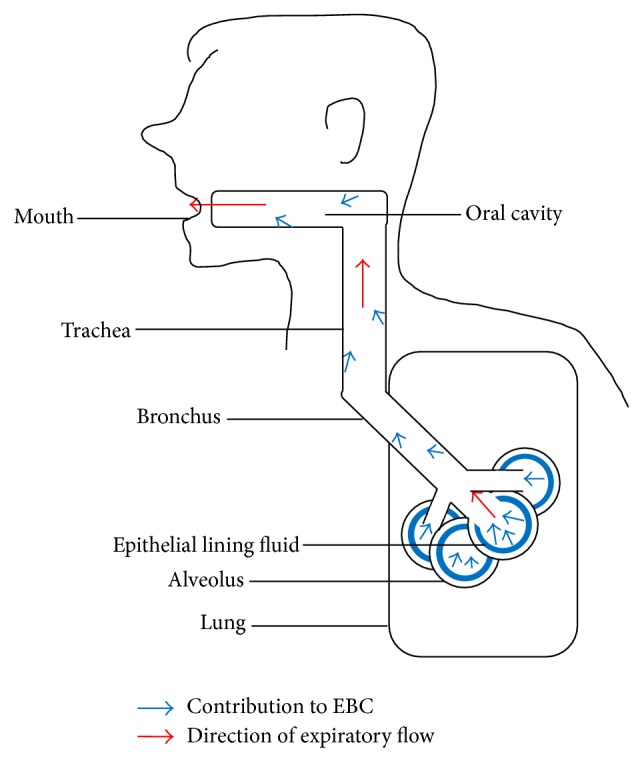
EBC consists of particles from ELF of alveoli, bronchi, and mouth, each with an unknown relative contribution.

As a result of the collection pathway, EBC also consists of nebulised fluid droplets from the alveoli, bronchi, and mouth, each with an unknown relative contribution (Figure 3). This nonsite specificity is a limitation, and it is inevitable that EBC of patients may consist of a fraction derived from areas not affected by the specific lung disease [67, 81]. EBC from lung cancer patients, for instance, will consist of a large fraction derived from nonmalignant areas. As EBC is collected through the mouth, saliva contamination is another potential problem. It can, however, be minimised by asking subjects to rinse their mouth prior to collection, swallowing accumulated saliva where possible [67] and routinely testing for salivary amylase in EBC samples [71].

While the volume of EBC is reproducible, levels of biomarkers in EBC may vary, and this gives rise to problems in repeatability and reproducibility [71, 81, 83]. This can, however, be overcome by concentrating samples, using assays with a low limit of detection and high sensitivity in many cases [71].

A range of biomarkers have been studied in EBC of COPD and lung cancer patients. The results are as shown in Table 1.

## 5. Plasma Proteomics

In addition to EBC, the serum protein profile is another easily collected yet cost-effective tool in detecting and monitoring lung cancer [9, 84, 85]. Elevated levels of C-reactive protein, serum amyloid A (SAA), mucin I, and *α*-I-antitrypsin can aid in distinguishing between healthy subjects or COPD patients [85] but are however low in sensitivity and/or specificity [86]. As such, novel markers are being described, such as the S100 proteins.

## 6. S100 Proteins

The S100 proteins are a family of more than 20 low molecular weight acidic proteins of 10–12 kDa which are calcium-binding, and they belong to the EF hand proteins subfamily [87–92]. They consist of two EF-hands with different calcium binding affinities joined together by a central hinge region [87, 91, 93]. This explains their role in regulating calcium-dependent intracellular processes [94] including protein phosphorylation, enzyme activity, cytoskeletal components, transcriptional factors, cell growth, and calcium homeostasis [87, 89, 90]. The S100 proteins can form homodimers, heterodimers, and oligomers with varying functions [87, 89, 90]. The majority of their coding genes are found on chromosome 1q21 which is frequently mutated [87, 95–97]. They have been implicated in many epithelial and soft tissue cancers including those of lung, breast, oesophagus, bladder, kidney, prostate, thyroid, gastric oral, colorectal, and liver [87, 95–97].

### 6.1. S100A8 and S100A9

S100A8 is also known as calgranulin A or myeloid-related protein 8 while S100A9 is also known as calgranulin B or myeloid-related protein 14. While much of the literature suggests that the S100A8 and S100A9 are proinflammatory, a body of research presents an opposing view. It is possible that the opposing effects of the calgranulins are concentration dependent, being proinflammatory at low concentrations and anti-inflammatory at high concentrations [98, 99]. 

A100A8 and S100A9 are believed to be anti-inflammatory by being preferentially oxidized, thereby scavenging ROS/RNS. Oxidative modifications by ROS/RNS and posttranslational modifications such as S-nitrosylation and S-glutathionylation are proposed to be the regulatory switches which activate such anti-inflammatory properties [98, 99]. 

**Figure 4:**
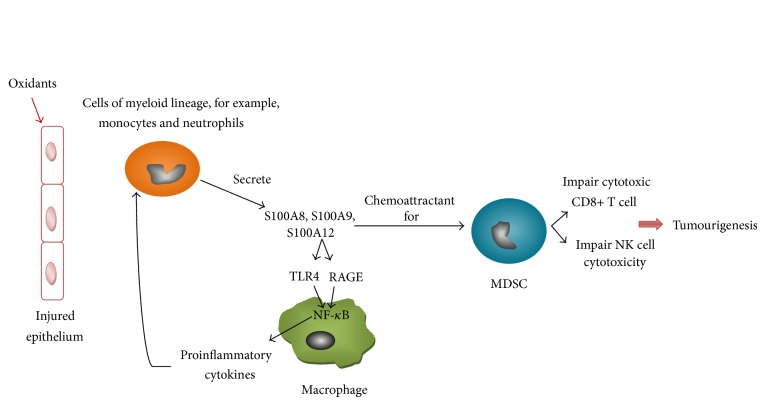
The calgranulins, S100A8, S100A9, and S100A12, are secreted by cells of the myeloid lineage such as neutrophils and monocytes. They bind to TLR4 and RAGE on macrophages and activate the NF-*κ*B signalling pathway which leads to the production of proinflammatory cytokines. The production of proinflammatory cytokines then provides a positive feedback by promoting the recruitment of more neutrophils and monocytes. S100A8 and S100A9 are also chemoattractants for MDSCs. MDSCs which move from bone marrow to peripheral blood cause immune suppression and enhance tumourigenesis by impairing cytotoxic CD8+ T cell and NK cell cytotoxicity.

Calgranulins S100A8 and S100A9, however, are also believed to play a role in inflammation by acting as chemokines for neutrophils and monocytes [88, 91, 100–102]. They reportedly bind to the receptor for advanced glycation end products (RAGE) and toll-like receptor-4 (TLR4) [88, 90, 103, 104]. This binding activates the NF-*κ*B transcription pathway, subsequent generation of downstream proinflammatory cytokines, and recruitment of inflammatory mediators such as neutrophils and monocytes in a positive feedback loop (Figure 4) [90, 103, 104]. As such, the S100 proteins have implicated many inflammation-related diseases including rheumatoid arthritis, juvenile idiopathic arthritis, cystic fibrosis, and chronic inflammatory bowel disease [88, 91, 93, 105–107]. Levels of S100A8 and S100A9 are elevated in the bronchoalveolar fluid of COPD patients compared to smokers, which suggest a potential as diagnostic markers of COPD [108]. Another study comparing acute respiratory distress syndrome (ARDS), cystic fibrosis (CF), and COPD suggests that S100A8 and S100A9 are linked to chronic inflammation while S100A12 is linked to acute inflammation [109]. 

Apart from playing a role in inflammation which promotes tumourigenesis (inflammation-induced cancer) [55], the S100 proteins are also capable of modulating host immune response to promote tumour progression [87]. 

 S100A8 and S100A9 are expressed by cells of myeloid origin, making up 40%–50% of their cytosolic content. Cells expressing S100A8 and S100A9 include granulocytes (e.g., neutrophils), monocytes, and early differentiation stages of macrophages [88, 93, 95, 97, 106, 110]. S100A12 is however only expressed in neutrophils [102, 111]. S100A8 and S100A9 predominantly function as heterodimer complex S100A8/A9 which is also known as calprotectin [88, 112]. Calprotectin is released by neutrophils and activated by monocytes, tumour cells, and myeloid-derived suppressor cells (MDSCs) [113]. It functions to regulate inflammation and inhibit myeloid cell differentiation [114]. 

MDSCs are precursors of macrophages, granulocytes, and dendritic cells [113] which increase in number during inflammation, cancer, and infection [115]. They suppress natural killer CD4+ and CD8+ T cell immunity against cancer by inhibiting dendritic cell differentiation to compromise antigen presentation (Figure 4) [112, 115–120]. MDSCs suppress this innate immunity through the induction of FOXP3+ T regulatory cells by secreting interleukin-10 (IL-10), interferon-gamma (IFN-*γ*) and high levels of ROS, peroxynitrite, and nitric oxide [116]. 

In tumorigenesis, MDSCs are attracted from bone marrow to peripheral blood by inflammatory cytokines (e.g., interleukin-1*β*, interleukin-6, prostaglandin E_2_), chemokines, tumour-derived growth factors, and myeloid-related proteins such as S100A9 and S100A8 [116, 117]. The production of proinflammatory S100A8/9 then sustains MDSC accumulation by an autocrine feedback through TLR4 and RAGE which activates the NF-*κ*B pathway and mitogen-activated protein kinase [113, 116, 117, 121]. Hence, similar to the positive feedback loop of oxidants, S100A8/A9 which is released by myeloid cells also promotes the recruitment of yet more leukocytes [122, 123].

S100A8/A9-positive myeloid cells are not only early infiltrating cells in the inflammatory process [97] but are also upregulated in epithelial malignancies including that of the prostate [124, 125], gastric [126], colon, and rectum [127, 128]. As such, S100A9 is suggested to be a potential marker in differentiating prostate cancer from benign prostate hyperplasia or healthy controls [125]. 

In lung cancer, a recent study found that the expression of S100A8 and S100A9 is increased in patients with NSCLC [116]. NSCLC patients with an overexpression of S100A9 are usually associated with poorly differentiated tumours [129, 130], lower 5-year survival rate [108], and higher rate of relapse [129]. Moreover, S100A9 in CD11b+CD14+ monocytic MDSC correlates with tumour response to platinum-based chemotherapy with low CD11b+CD14+S100A9+ having longer progression-free survival [116]. These suggest the possibility of S100A8 and S100A9 as prognostic markers of NSCLC. 

Lastly, S100A8 and S100A9 also play a role in cell proliferation and metastasis of primary tumours into the lung [87]. Their expression is increased in pulmonary myeloid and endothelial cells through the production of vascular endothelial growth factor-A, transforming growth factor-*β*, and TNF-*α* by primary tumours before metastasis occurs [87, 131, 132]. S100A8 and S100A9 not only promote the recruitment of CD11b+ myeloid cells but also act as chemoattractants which draw tumour cells to premetastatic sites in the lungs [87, 110]. They recruit CD11b+ myeloid cells by activating p38 mitogen-activated protein kinase (MAPK) which promotes migration [110]. SAA3, which is induced by S100A8, interacts with TLR4 to stimulate the NF-*κ*B pathway in promoting CD11b+ myeloid cell accumulation [110, 133]. In addition, S100A8 and S100A9 also increase cancer cell motility through p38-mediated activation of pseudopodia [87, 131]. This makes S100A8/A9 a potential target for inhibiting lung metastasis. 

## 7. Future Directions

*Early Diagnosis, Predicting Prognosis, and Personalised Medicine. *EBC and serum are noninvasive and minimally invasive techniques which are cost effective and easily sampled. If EBC markers of oxidative stress and serum proinflammatory S100 proteins or other candidate entities are diagnostic for COPD and NSCLC, it could greatly improve survival outcome by allowing early diagnosis and thus treatment. 

As many NSCLC patients do not behave as predicted based on tumour staging, new markers are also needed to more accurately predict prognosis [134]. Prognostic biomarkers indicative of metastatic potential, response to treatment, and patient survival could aid in deciding treatments. For example, using CD11b+CD14+S100A9+ to predict response to chemotherapy could be used to decide if patients should be given adjuvant or neoadjuvant chemotherapy or any chemotherapy at all. 

Furthermore, it will be beneficial to discover more specific and sensitive serum biomarkers for lung cancer as well as to personalise anticancer therapies. For instance, knowing the reduced overall survival of patients with an overexpression of S100A9 may not only identify patients who are at high risk of a poor outcome [134] but also allow the administration of personalised anticancer therapy which targets S100A9 specifically to optimise outcome [64].

The S100 proteins have a great potential to be the new diagnostic tumour markers, prognostic predictor, and possibly therapeutic targets for NSCLC.

## Figures and Tables

**Table 1:** Summary of EBC markers of oxidative stress and antioxidant capacity including S100 proteins in COPD and lung cancer (legend: “↑”: elevated, “↓”: decreased, “*≈*”: no difference, “×”: undetectable).

Category	Biomarkers	COPD patients		Lung cancer patients	
		EBC (compared to healthy volunteers)	EBC (compared to smokers or ex-smokers)	EBC (compared to healthy volunteers)	EBC (compared to specific controls)
Markers of oxidative stress					
Reactive oxygen species	Hydrogen peroxide	↑ [34, 135–138]	↑ [34]	↑ [135]	

Reactive nitrogen species	Nitric oxide	↑ [139–144]	↑ [139, 141]	↑ [140]	↑ (controls = cancer patients) [142]
	Nitrite	↑ [145]	↑ [145]		↑ (controls = cancer patients) [142]
	Nitrate	High variability [146]			↓ (controls = cancer patients) [142]
		*≈* [141, 147]			
	Peroxynitrite	↑ [148, 149]	↑ [148, 149]		

Lipid peroxidation products/eicosanoids (arachidonic acid derivatives)	8-isoprostane	↑ [137, 148, 150, 151]	↑ [34, 148, 150]		↑ [135]
					*≈* (controls = healthy smokers) [152]
	Malondialdehyde	↑ [138, 153]	↑ [153]	↑ [154]	
		*≈* [137, 155]	*≈* [137]		
	Leukotrienes B_4_	↑ [156–158]	↑ [159]		↑ (controls = patients without pulmonary disease) [156]
	Leukotriene C4			↑ [160]	
	Leukotriene D4			↑ [160]	
	Leukotriene E4	*≈* [158]		↑ [160]	
	Prostaglandin E_2_	↑ [158]			
	Thromboxane B2 (the stable form of thromboxane A2)	↓ [158]			
	Prostaglandin D2-methoxime	*≈* [158]			
	Prostaglandin F2*α*	↑ [158]			

Cytokines and proteins	Tumour necrosis Factor-*α*	*≈* [47]		↑ [161]	↑ (controls = smokers without COPD or lung cancer) [152]
	Interleukin-6	↑ [162]		↑ [163]	
		*≈* [47]			
	Interleukin-8	*≈* [47, 164]	*≈* [164]		↑ (controls = patients without pulmonary diseases) [156]
	Metaloproteinase-9		↑ [134]		↑ (controls = patients without pulmonary diseases) [165]
	Vascular endothelial growth factor				↑ (controls = healthy smokers) [152]
	Endothelin-1	↑ [166]		↑ [167]	
				↑ [168]	

Volatile organic compounds	Alkanes, alkane derivatives, benzene derivatives	↑ (exhaled ethane) [169]		↑ [170, 171]	

Heme breakdown product	Carbon monoxide	↑ [144]	↑ [144]		

pH		↓ [164, 172–175]	*≈* [173]	*≈* [172]	

Deoxyribonucleic acid mutations	3p microsatellite alterations			↑ [176]	
	Tumour suppressor gene P53 mutations			↑ [177]	
	Oncogene KRAS			↑ [178]	
	Epidermal growth factor receptor (EGFR) gene mutations			↑ (in small number of heavy smokers with squamous cell carcinoma) [179]	
	Gene promoter methylation mutations			↑ [180]	
	Mitochondrial DNA mutations				↑ (controls = smokers, exsmokers without chronic respiratory diseases, respiratory illnesses, or lung cancer) [70]

Viruses	Human papilloma virus				↑ (controls = patients suspected of lung cancer but with negative cytology) [181]

Markers measuring antioxidant capacity					
Enzymes	Superoxide dismutase				↑ (controls = patients without pulmonary diseases) [182]

Nonenzymatic antioxidants	Ascorbic acid/vitamin C			↓ (↑ in percentage degradation/oxidation rate) [161]	
	Urate	× [175]	× [175]		
	Ferritin				↑ (controls = patients affected by transudative pleural effusion and without pulmonary diseases) [182]
	Bilirubin	*≈* [175]	*≈* [175]		
